# Light and temperature effects on miR156 transgenic switchgrass flowering: A simulated latitudinal study

**DOI:** 10.1002/pld3.26

**Published:** 2017-11-03

**Authors:** Chelsea R. Johnson, Reginald J. Millwood, Zeng‐Yu Wang, Charles N. Stewart

**Affiliations:** ^1^ Department of Plant Sciences University of Tennessee Knoxville TN USA; ^2^ BioEnergy Science Center Oak Ridge National Laboratory Oak Ridge TN USA; ^3^ Noble Research Institute Ardmore OK USA

**Keywords:** flowering time, plant breeding and biotechnology, regulatory RNA/noncoding RNA, switchgrass, transcriptional regulation/regulation of transcription—general

## Abstract

The control of flowering in perennial grasses is an important trait, especially among biofuel feedstocks. Lignocellulosic biomass may be increased commensurate with decreased or delayed flowering as the plant allocates energy for stems and leaves harvested for bioenergy at the end of the growing season. For transgenic feedstocks, such as switchgrass (*Panicum virgatum* L.) grown in its geographic center of distribution, it is foreseeable that regulators may require greatly decreased gene flow frequencies to enable commercialization. Transgenic switchgrass with various overexpression levels of a rice microRNA gene, miR156, when grown in field conditions, holds promise for decreased flowering, yielding high biomass, and altered cell wall traits, which renders it as a potential crossing partner for further breeding with switchgrass lines for decreased recalcitrance. In the current research, we simulated a latitudinal cline in controlled growth chamber experiments for various individual sites from the tropics to cool‐temperate conditions which included weekly average high and low temperatures and day lengths over the switchgrass growing season for each simulated site: Guayaquil, Ecuador; Laredo, Texas, USA; and Brattleboro, Vermont, USA. Flowering and reproduction among transgenic lines with low (T‐14 and T‐35)‐to‐moderate (T‐27 and T‐37) overexpression of miR156 were assessed. Lower simulated latitudes (higher temperatures with low‐variant day length) and long growing seasons promoted flowering of the miR156 transgenic switchgrass lines. Tropical conditions rescued the flowering phenotype in all transgenic lines except T‐27. Higher numbers of plants in lines T‐35 and T‐37 and the controls produced panicles, which also occurred earlier in the study as temperatures increased and day length decreased. Line T‐14 was the exception as more clonal replicates flowered in the cool‐temperate (Vermont) conditions. Increased biomass was found in transgenic lines T‐35 and T‐37 in tropical conditions. No difference in biomass was found in subtropical (Texas) chambers, and two lines (T‐14 and T‐35) produced less biomass than the control in cool‐temperate conditions. Our findings suggest that switchgrass plants engineered to overexpress miR156 for delayed flowering to promote bioconfinement and biomass production may be used for plant breeding at tropical sites.

## 1 INTRODUCTION

Plants heavily depend on endogenous cues, photoperiod, and temperature to correctly time their change from vegetative to reproductive state (Franklin, 2009; Penfield, 2008; Srikanth & Schmid, 2011). Cultivars of switchgrass (*Panicum virgatum* L.), a cellulosic biofuel feedstock candidate, are divided into either lowland or upland ecotypes based on latitudinal origin (Casler, Vogel, Taliaferro, & Wynia, 2004; Porter, 1966). The cultivation of switchgrass ecotypes more than one USDA hardiness zone north or south of their adaptive zone can affect their flowering, vigor, and survival because of a changes in day length, temperature, and other factors (Casler, 2005; Hopkins, Vogel, Moore, Johnson, & Carlson, 1995; Kiniry et al., 2013; Wullschleger, Davis, Borsuk, Gunderson, & Lynd, 2010). Because environmental conditions are such a strong cue for flowering, it stands to reason that switchgrass plants genetically engineered for delayed flowering might have altered flowering phenology depending on latitude and environments associated with field sites therein.

There are numerous examples of temperature or photoperiod effects on flowering. Balasubramanian, Sureshkumar, Lempe, and Weigel (2006) showed that a 2–4°C increase in growing temperature was just as effective at flower induction as a change in day length for *Arabidopsis thaliana*. Flowering in *Arabidopsis* is normally inhibited in a short‐day cycle, but plants flowered at approximately the same rate in short‐day periods at 25 or 27°C as *Arabidopsis* plants being grown in long‐day cycles at 16°C in growth chambers (Balasubramanian et al., 2006). A review by McClung, Lou, Hermand, and Kim (2016) surveyed temperature effects on flower initiation; the effects can be mediated or confounded by temperature stress conditions. When various plant species were examined for environmental effects of reproductive timing, Sherry et al. (2007) found that field‐grown switchgrass in Oklahoma had accelerated flowering under a 4°C increase in growing temperature, which was further exacerbated with increased water availability. Some switchgrass cultivars flower the same time each year regardless of temperature differences which suggests that switchgrass may be more sensitive to photoperiod than some other environmental factors (Hopkins et al., 1995; Sanderson & Wolf, 1995; Van Esbroeck, Hussey, & Sanderson, 2003). Indeed, studies have shown a change in flower initiation due to altered photoperiods in both upland (Castro, Boe, & Lee, 2011) and lowland (Alexander, Haynes, Burris, Jackson, & Stewart, 2014; Van Esbroeck et al., 2003) switchgrass cultivars. In addition to photoperiod, Alexander et al. (2014) also examined the effects of temperature and plant growth regulators, namely auxin and gibberellin, on switchgrass flowering.

Besides exogenous cues, there are genetic determinants of flower timing. These have been studied recently using reverse genetics experiments. Switchgrass has been genetically engineered for altered flowering phenotypes. When miR156 was overexpressed in switchgrass (cv. “Alamo”), the level of expression appeared to convey several phenotypic effects, including altered flower time (Chuck et al., 2011; Fu et al., 2012). Depending on the transgenic event, the plant biomass, architecture, and flowering time ranged from undiscernible from the nontransgenic parent to nonflowering dwarf plants when miR156 was highly overexpressed (Fu et al., 2012).

From a bioenergy feedstock perspective, the desirable phenotype is maximal biomass production with low inputs, decreased‐to‐inhibited flowering, and cell walls that are readily converted to sugars. While delayed/nonflowering phenotype would be beneficial from a transgenic‐regulatory standpoint in that gene flow would be decreased (Kausch et al., 2010; Sang, Millwood, & Stewart, 2013), plant breeders would likely need some sexual reproductive capacity for conventional switchgrass improvement, that is, seed production and the establishment of commercial fields (McLaughlin & Kszos, 2005; Wolfe & Fiske, 1995). Fu et al. (2012) performed a greenhouse experiment mimicking summertime cool‐temperate conditions: (16‐hr days, 26°C average temperature), but it is possible that a change in temperature, day length, or a combination of the two could reinstate a flowering phenotype suitable for seed production.

To test this hypothesis, switchgrass plants genetically engineered to overexpress miR156 (Fu et al., 2012), a regulatory microRNA that is involved in the flower induction pathway, were grown in growth chambers that simulated the day lengths, temperatures, and season length of specific sites that were largely selected at latitudes outside of the adaptation zone of “Alamo” switchgrass. While such growth chamber simulations cannot replicate field conditions at the target site, the experiments should be valuable to give first‐order comparisons. Throughout each growing season, the growth chamber settings were based on the average weekly day length and high/low temperatures of representative areas that included tropical (Guayaquil, Ecuador), subtropical (Laredo, Texas, USA), and cool temperate (Brattleboro, Vermont, USA) (Table 1). The high temperature and constant 12‐hr (short, for switchgrass) day length of the tropical growing conditions resulted in panicle production in the control and all but one of the transgenic lines. It is possible that switchgrass plants with delayed or nonflowering phenotypes could be grown for breeding purposes in tropical climate conditions for seed production based on flower initiation in tropical growth chamber conditions.

**Table 1:** The minimum, maximum, and average season length, temperature, and photoperiod settings for each of the three growth chamber experiments. Temperature and day length settings were changed weekly to mimic seasonal changes

	Tropical (Guayaquil, Ecuador)	Subtropical (Laredo, Texas)	Cool‐ temperate (Brattleboro, Vermont)
Growth Season Length			
	52 Weeks	41 Weeks	23 Weeks
Temperature (Day/Night °C)			
Minimum	33/25	20/14	16/14
Maximum	33/25	40/26	29/17
Average	33/25	33/21	24/14
Photoperiod (hr: min)			
Minimum	12:00	10:37	11:47
Maximum	12:00	13:52	15:20
Average	12:00	12:34	14:11

## 2 MATERIALS AND METHODS

### 2.1 Plants, experimental design, and growth conditions

The miR156 low overexpression lines T‐14 and T‐35, medium overexpression lines T‐27 and T‐37, and one nontransgenic line from Fu et al. (2012) were used for each of the growth chamber experiments. All lines originated from the lowland switchgrass cultivar ‘Alamo’, and transgenic lines have been characterized and described previously in the greenhouse (Fu et al., 2012) and a Knoxville, Tenn., USA field (Baxter et al., 2017). Plants were grown in Percival PCG‐15 growth chambers (Percival Scientific, Perry, Iowa USA) with temperature and photoperiod settings that corresponded to tropical, subtropical, or cool‐temperate growing conditions from published day length and temperature highs and lows for each day of their respective growing seasons (Table 1; Table S1). Typically, the lowland switchgrass growing season begins with vegetative flushes, which occur when weekly average temperatures are above 15/10°C for day/night, and ends when weekly minimum temperatures average below 15°C (Gu, Wylie, & Howard, 2015; Sanderson & Wolf, 1995). All experiments were started on the same day. Plants were culled to three tillers per pot, cut back to 20.32 cm, and grown in 12‐L pots. Each growing condition was replicated in two growth chambers, and four clones of each line were randomly placed in each chamber (5 lines × 4 clones = 20 plants/chamber; Fig. S1a). The pot was the experimental unit. Pot locations within the chamber were randomized again at mid‐season to avoid any positional growing effects (Fig. S1b). Plants were watered one to three times per week and fertilized with Peters 20‐20‐20 fertilizer (J.R. Peters Inc., Allentown, Penn. USA) once every two weeks.

### 2.2 Plant characterization

The date for first flower emergence of each plant was recorded, and panicles were counted and removed throughout the growing season. Plant height was measured from the level of potting mix to the tallest point of the plant. The two tallest tillers were used to measure leaf length and width, node number, and internode diameter. The flag leaf or topmost mature leaf was used for length and width measurements. Internode diameter was measured using a Maxwell 150‐mm digital caliper between the third and fourth nodes from the potting‐mix level. All but 10 cm of aboveground biomass was harvested at the end of the experiment. The biomass by pot was placed in a drying oven at 43°C for 300 hr prior to taking dry weight data. Tillers were tallied at harvest.

### 2.3 Statistical analysis

Statistical analysis was performed using SAS version 9.4 (SAS Institute Inc., Cary, NC). Tiller number counts observed under tropical and subtropical conditions did not meet assumptions for equal variances or normal distributions. Therefore, these data were log‐transformed to satisfy these requirements. To determine whether any statistical differences existed among transgenic plant lines and the nontransgenic parent, a one‐way ANOVA was performed for each experimental condition. If any ANOVA indicated a statistical difference at the 0.05 level, then mean separation tests were performed first using Fisher's least significant difference (LSD) with lines reported as different if the *p*‐value was less than .05.

## 3 RESULTS AND DISCUSSION

### 3.1 Flowering phenotype

**Figure 1:**
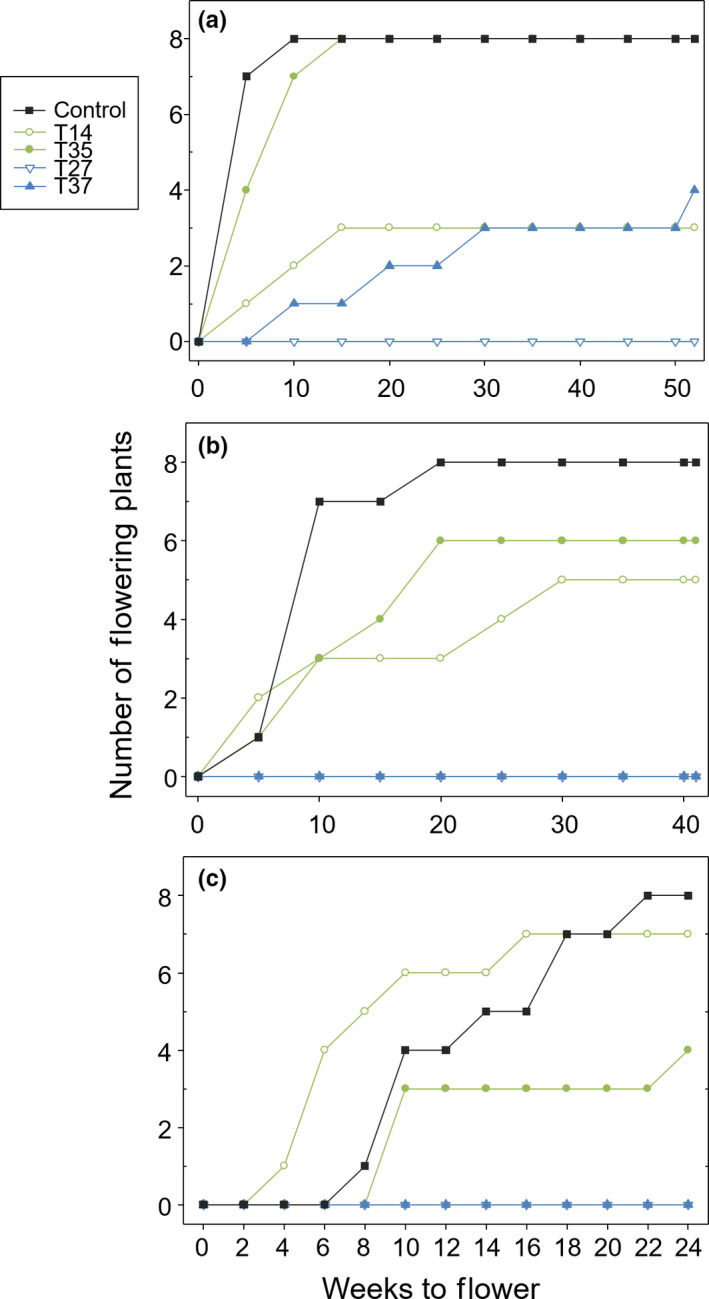
Time to first flower and number of plants flowering throughout the (a) tropical, (b) subtropical, and (c) cool‐temperate growing seasons. Lines labeled in green (T‐14 and T‐35) represent low miR156 overexpression, and lines labeled in blue (T‐27 and T‐37) represent medium miR156 overexpression

The nontransgenic control and low miR156 overexpression line T‐35 were the only lines to flower under all three growing conditions. The medium overexpression line T‐27 did not produce panicles under the growth chamber conditions tested, as previously observed in greenhouse experiments (Fu et al., 2012) and during a three‐year field experiment in Knoxville, Tenn., USA (Baxter et al., 2017). These findings suggested that the nonflowering phenotype resulted from relatively high miR156 overexpression in both settings (Baxter et al., 2017). The tropical regime was the only one in which all lines, excluding T‐27, produced panicles. However, line T‐14 produced so few panicles that the average panicle number per plant was statistically zero (Table 2). Switchgrass flowering time appears to be more dependent on photoperiod than temperature (Sanderson & Wolf, 1995); therefore, the constant 12‐hr day length in the tropical growth chambers may be responsible for more lines flowering compared to the subtropical and cool‐temperate growth chambers, which had longer days during the growth season. The earliest flowering time was observed in subtropical‐condition growth chambers, which was most similar to where ‘Alamo’ would be cultivated in the field; all flowering lines produced panicles by week five (Figure 1b). The high ambient temperature of the subtropical conditions most likely promoted flowering, especially during the short photoperiods in the beginning of the season (Table S1; Li, Li, Liu, & Liu, 2016). Short‐day plants such as switchgrass have shown accelerated flowering when treated with warmer temperatures (Alexander et al., 2014; Cleland, Chlarlello, Loarle, Mooney, & Field, 2006; Hartman & Nippert, 2013; Sherry et al., 2007; Van Esbroeck et al., 2003). The long day‐lengths of the subtropical chambers may have also contributed to flowering despite miR156 overexpression. When *Arabidopsis* was engineered to overexpress miR156, transgenic plants did not flower under short‐day conditions until 7 months after the start of the experiment. However, when plants were exposed to long‐day conditions, only a moderate delay in flowering was observed compared to controls (Schwab et al., 2005).

**Table 2:** Phenotypic characterization of miR156 transgenic switchgrass plants under tropical, subtropical, and cool‐temperate growth chamber settings

	Panicle number	Tiller number	Plant height (cm)	Leaf length (cm)	Leaf width (cm)	Node number	Internode diameter (mm)
Tropical							
Control	25 ± 3.1^a^	31 ± 2.5^c^	169.3 ± 4.5^a^	28.1 ± 4.3^ab^	0.6 ± 0.1^ab^	10 ± 0.8^b^	2.35 ± 0.15^a^
T‐14	0 ± 0.2^b^	6 ± 1.0^d^	101.4 ± 14.8^b^	26.0 ± 5.9^ab^	0.7 ± 0.1^a^	7 ± 1.6^b^	1.66 ± 0.20^b^
T‐35	18 ± 2.6^a^	68 ± 6.3^b^	157.1 ± 3.7^a^	39.5 ± 2.4^a^	0.8 ± 0.1^a^	7 ± 0.4^b^	2.74 ± 0.14^a^
T‐27	0^b^	194 ± 41.2^a^	113.1 ± 8.7^b^	24.9 ± 1.5^b^	0.3 ± 0.04^b^	8 ± 0.3^b^	0.81 ± 0.14^b^
T‐37	3 ± 2.0^b^	226 ± 24.2^a^	146.3 ± 5.9^a^	18.7 ± 1.4^b^	0.3 ± 0.04^b^	13 ± 0.8^a^	1.36 ± 0.15^b^
Subtropical							
Control	13 ± 2.0^a^	30 ± 2.9^c^	163.4 ± 6.4^a^	28.9 ± 3.1^a^	0.8 ± 0.1^a^	11 ± 0.7^a^	3.61 ± 0.16^ab^
T‐14	2 ± 1.0^b^	16 ± 3.2^d^	118.7 ± 13.0 ^cd^	30.8 ± 2.9^a^	0.8 ± 0.1^a^	8 ± 0.7^ab^	2.53 ± 0.15^bc^
T‐35	3 ± 1.1^b^	48 ± 7.9^b^	154.9 ± 3.9^ab^	32.1 ± 3.3^a^	0.8 ± 0.1^a^	8 ± 0.5^ab^	3.91 ± 0.14^a^
T‐27	0^b^	168 ± 34.6^a^	97.2 ± 5.8^d^	27.1 ± 1.3^a^	0.2 ± 0.02^b^	7 ± 0.3^b^	0.66 ± 0.09^d^
T‐37	0^b^	161 ± 16.8^a^	128.8 ± 6.9^bc^	26.4 ± 3.3^a^	0.5 ± 0.04^b^	9 ± 0.6^ab^	1.42 ± 0.04 ^cd^
Cool temperate							
Control	2 ± 0.6^ab^	24 ± 2.2^c^	160.9 ± 3.9^a^	65.2 ± 1.8^a^	1.3 ± 0.04^a^	5 ± 0.3^ab^	4.62 ± 0.21^a^
T‐14	3 ± 0.7^a^	18 ± 2.2^c^	128.8 ± 4.7^b^	42.5 ± 1.4^c^	1.1 ± 0.1^b^	5 ± 0.2^a^	3.96 ± 0.17^ab^
T‐35	1 ± 0.2^bc^	17 ± 2.2^c^	139.2 ± 5.5^b^	56.6 ± 1.3^b^	1.2 ± 0.04^ab^	4 ± 0.2^b^	4.07 ± 0.23^a^
T‐27	0^c^	196 ± 8.2^a^	101.8 ± 3.2^c^	26.6 ± 1.0^d^	0.3 ± 0.02^d^	5 ± 0.2^a^	1.20 ± 0.07^c^
T‐37	0^c^	69 ± 3.4^b^	137.0 ± 3.4^b^	45.2 ± 1.8^c^	0.8 ± 0.03^c^	5 ± 0.2^a^	2.97 ± 0.10^b^

All data were taken at the end of the respective season. The topmost leaf was used to measure leaf blade length and width, and internode 3 was used for internode diameter. Two tillers were measured for each replicate. Values are mean ± *SE* (*n* = 8). Letters indicate significant differences at *p* < .05, Fisher's LSD for log‐transformed (tropical: tiller number; subtropical: tiller number) and nontransformed (all other measurements) data.

The control was the only line to have all plants transition to the reproductive stage in subtropical conditions, and all replicates of the control and line T‐35 began flowering by week 15 in the tropical chambers (Figure 1a,b). The control was also the only line in which all replicates flowered in the cool‐temperate experiment (Figure 1c).

Although some data cannot be directly compared among experiments because of differences in season length (Fig. S2; Table S1), it is interesting to note that the average number of panicles was higher in the tropical and subtropical experiments (short days) than the cool temperate (long days) for all flowering lines except T‐14 (Table 2). In general, switchgrass is thought to be a facultative short‐day plant (Alexander et al., 2014; Porter, 1966; Van Esbroeck et al., 2003), but there is evidence suggesting upland cultivars may have a long‐day flowering response (Casler, 2012; Castro et al., 2011). The increase in panicle number, combined with other phenotypic traits of line T‐14 grown under cool‐temperate conditions, suggests that T‐14 may behave more like an upland switchgrass ecotype.

### 3.2 Biomass and phenotypes

**Figure 2:**
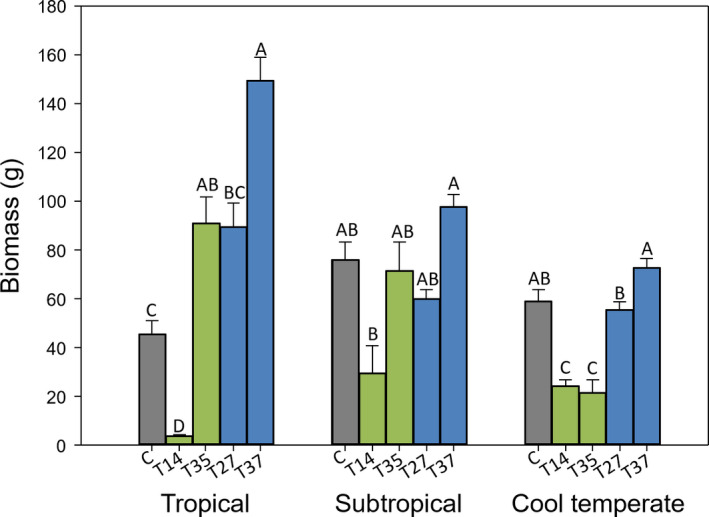
Biomass production per pot of miR156 transgenic switchgrass and control plants grown in tropical, subtropical, or cool‐temperate conditions. Error bars represent *SE*. Letters denote statistical differences within each growth condition at *p *=* *.05, Fisher's LSD

When grown under tropical temperature and day length settings, transgenic lines T‐35 and T‐37 produced twofold and threefold more biomass than the control, respectively (Figure 2). The high biomass yield was most likely because of the increased tiller number of both T‐35 and T‐37 (Table 2), which could have been driven by high temperature (Hartman & Nippert, 2013; Kandel, Wu, & Kakani, 2013). Therefore, there is a vegetative effect of reprogramming flowering as well as flowering itself, that is, increased tillering as one example, which is a pleiotropic effect observed from overexpressing miR156 in switchgrass (Baxter et al., 2017; Chuck et al., 2011; Fu et al., 2012), red clover (Zheng, Liu, Goff, Dinkins, & Zhu, 2016), as well as other species (reviewed in Trumbo, Zhang, & Stewart, 2015). No differences were observed in biomass production in the subtropical‐simulation experiment compared to control plants (Figure 2). None of the four transgenic lines produced significantly more biomass than the control under cool‐temperate conditions, but both low miR156 overexpression lines (T‐14 and T‐35) produced significantly less biomass (Figure 2).

While none of the transgenic lines were taller than controls, lines T‐14 and T‐27 were significantly shorter in all growth conditions (Table 2). Line T‐27 was shorter than controls in both subtropical and cool‐temperate conditions as was observed in both greenhouse and field conditions (Baxter et al., 2017; Fu et al., 2012). Line T‐37 was shorter in both subtropical and cool‐temperate conditions, and line T‐35 was shorter in cool‐temperate settings (Table 2). These data are in contrast to field experiments in Tennessee, in which line T‐35 plants were largest after three growing seasons (Baxter et al., 2017). Leaf length differed significantly in the cool‐temperate experiment with all transgenic leaves being shorter than the control (Table 2), and this was the first time a difference in leaf length was reported for the transgenic lines (Fu et al., 2012). For leaf width, differences were found in subtropical and cool‐temperate conditions. Lines T‐27 and T‐37 leaves had smaller widths than the control in both conditions, and T‐14 leaf widths were smaller than the control only in the cool‐temperate experiment (Table 2). These results suggest that perhaps the constant 12‐hr days coupled with warm temperatures in the simulated tropics resulted in wide leaf production as Fu et al. (2012) also reported a decrease in leaf width for medium overexpression lines. Node number did not differ between lines when grown in cool‐temperate settings, but T‐37 and T‐27 had significantly more nodes than the control in tropical and subtropical conditions, respectively (Table 2). The internode diameter of line T‐35 did not differ from the control in any of the experimental settings, but medium overexpression lines T‐27 and T‐37 had tillers with decreased diameter than the control in all conditions. Line T‐14 internode diameter was smaller than the control only in the tropical experiment (Table 2).

## 4 CONCLUSION

These experiments show that simulated latitudinal differences result in altered switchgrass phenotypes among lines genetically engineered for delayed flowering. If switchgrass plants overexpressing miR156 were grown in the tropics with invariant day length and relatively constant high temperatures, such as coastal Ecuador, such a location could be used for breeding as flowering and seed production may be possible.
