# Supplementary material for: Light and temperature effects on miR156 transgenic switchgrass flowering: A simulated latitudinal study
Source: Plant Direct. 2017 Nov 3;1(5):e00026. doi: 10.1002/pld3.26 (PMC6508523; doi:10.1002/pld3.26)
Supplement: Supplementary file 1 [file PLD3-1-e00026-s001.pdf]

Supplemental Material

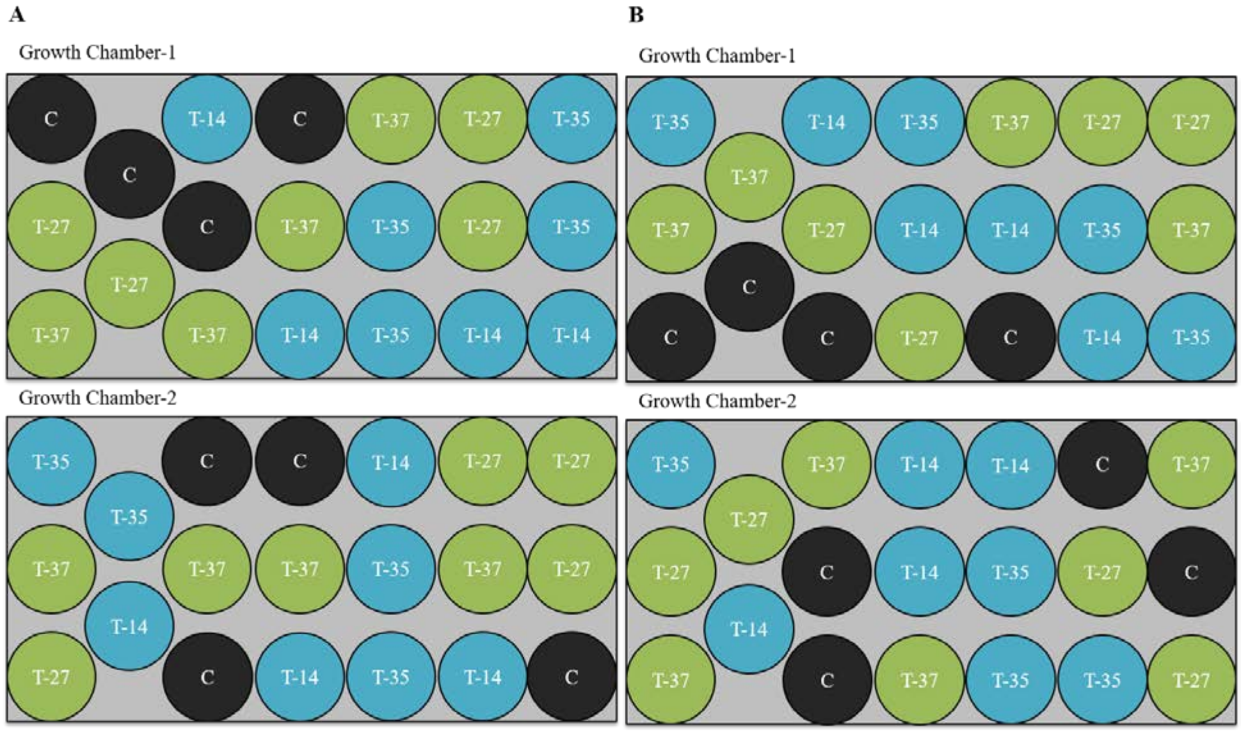

Supplemental Figure 1. Growth chamber study with a randomized complete block design. Each experiment was replicated in two growth chambers, and each growth chamber contained four replicates of each line. Lines are color-coded, which include a nontransgenic control (C) shown in black, low overexpression lines T-14 and T-35 (blue), and two medium overexpression lines T-27 and T-37 (green). A) Arrangement of pots from beginning of the season to mid-season. B) Pots were re-arranged in a different randomized design from mid-season to end of season.

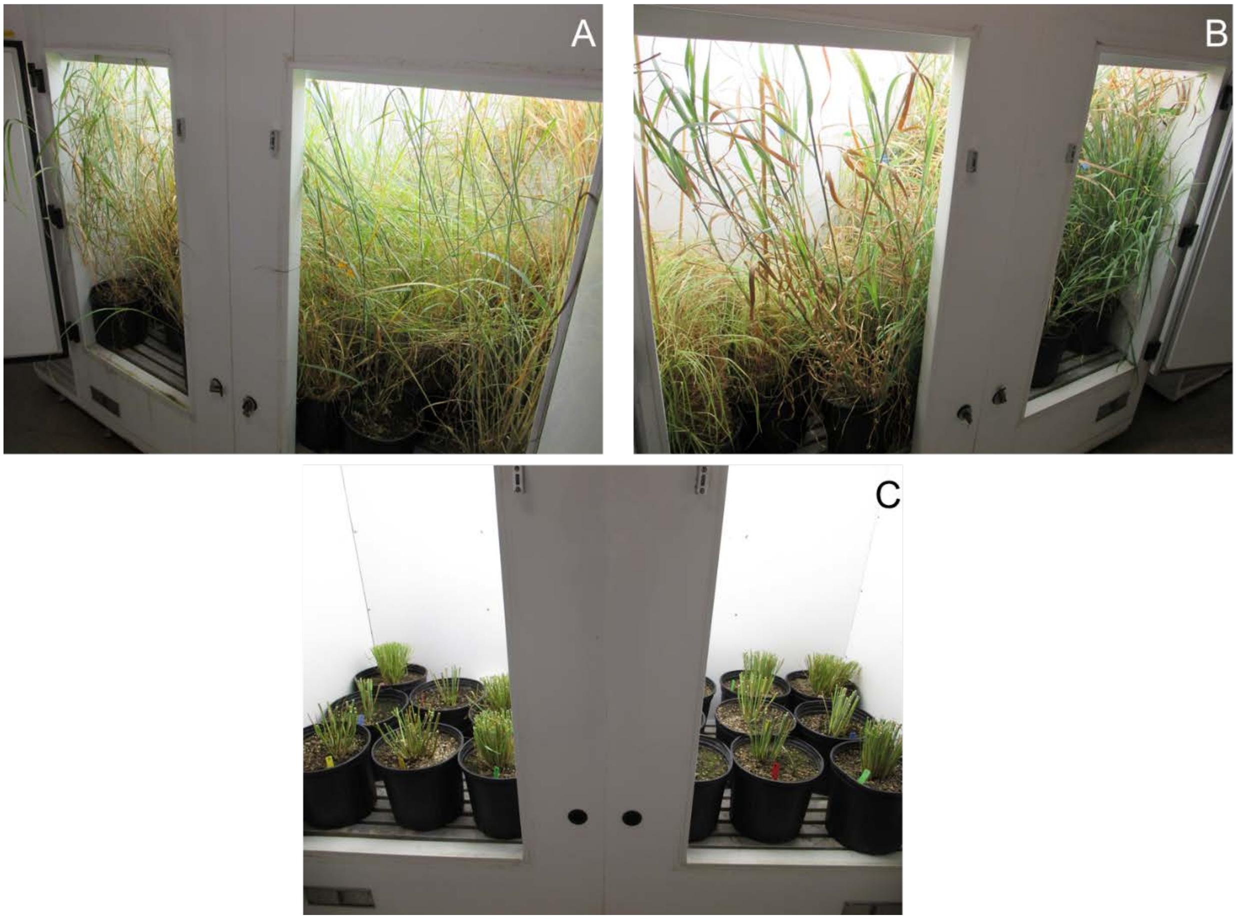

Supplemental Figure 2. Switchgrass lines overexpressing miR156 at low (T14 and T35) and medium (T27 and T37) levels and nontransgenic ‘Alamo’ control plants grown in three different climate simulations just before harvest (A and B) and after aboveground biomass harvest (C). A) Control and miR156 transgenic switchgrass grown in tropical temperature and day-length settings produced tall tillers that were subject to lodging. B) Plants grown in sub-tropical conditions produced thicker tillers that grew into the lights of the growth chamber in some cases. C) A cool-temperate growth chamber after plant biomass was harvested.

Supplemental Table 1. Growth chamber regimes simulated sub-tropical and cool temperate conditions. Tropical settings were static the entire 52 week period (35/25°C day/night temperature, 12:00 hr day length). Some night temperatures reached below 14°C (highlighted), but no settings below 14°C were used due to growth chamber setting restrictions.

**Sub-tropical (Laredo, TX, USA)**			**Cool temperate (Brattleboro, VT, USA)**		
Week	Avg. Temp. (D/N) ˚C	Day length	Week	Avg. Temp. (D/N) ˚C	Day length
1	21/9	11:18	1	16/3	14:00
2	26/14	11:28	2	20/6	14:18
3	25/13	11:40	3	21/8	14:34
4	24/11	11:52	4	21/12	14:48
5	27/14	12:03	5	25/13	15:00
6	31/17	12:15	6	22/10	15:09
7	28/17	12:26	7	23/11	15:16
8	32/19	12:37	8	26/12	15:20
9	33/18	12:49	9	27/14	15:20
10	31/18	12:59	10	29/17	15:17
11	34/19	13:09	11	29/16	15:11
12	32/20	13:19	12	29/17	15:01
13	34/20	13:28	13	27/15	14:50
14	32/19	13:35	14	27/15	14:36
15	35/23	13:42	15	27/14	14:20
16	37/24	13:47	16	25/13	14:04
14	37/24	13:50	14	25/12	13:46
18	38/26	13:52	18	27/13	13:27
19	38/26	13:52	19	26/15	13:07
20	36/26	13:51	20	23/9	12:48
21	38/25	13:47	21	20/6	12:28
22	36/25	13:42	22	19/7	12:07
23	37/25	13:36	23	20/10	11:47
24	39/26	13:29			
25	40/26	13:20			
26	40/26	13:11			
27	39/26	13:01			
28	38/26	12:51			
29	39/26	12:40			
30	37/25	12:29			
31	38/25	12:17			
32	35/23	12:06			
33	33/23	11:55			
34	34/22	11:44			
35	33/22	11:32			
36	33/21	11:22			
37	31/20	11:11			
38	30/19	11:01			
39	30/18	10:52			
40	27/17	10:44			

41	20/12	10:37
